# matchRanges: generating null hypothesis genomic ranges via covariate-matched sampling

**DOI:** 10.1093/bioinformatics/btad197

**Published:** 2023-04-21

**Authors:** Eric S Davis, Wancen Mu, Stuart Lee, Mikhail G Dozmorov, Michael I Love, Douglas H Phanstiel

**Affiliations:** Curriculum in Bioinformatics and Computational Biology, University of North Carolina at Chapel Hill, Chapel Hill, NC, United States; Department of Biostatistics, University of North Carolina at Chapel Hill, Chapel Hill, NC, United States; Genentech, South San Francisco, CA, United States; Department of Biostatistics, Virginia Commonwealth University, Richmond, VA, United States; Department of Pathology, Virginia Commonwealth University, Richmond, VA, United States; Curriculum in Bioinformatics and Computational Biology, University of North Carolina at Chapel Hill, Chapel Hill, NC, United States; Department of Biostatistics, University of North Carolina at Chapel Hill, Chapel Hill, NC, United States; Department of Genetics, University of North Carolina at Chapel Hill, Chapel Hill, NC, United States; Lineberger Comprehensive Cancer Center, University of North Carolina at Chapel Hill, Chapel Hill, NC, United States; Curriculum in Bioinformatics and Computational Biology, University of North Carolina at Chapel Hill, Chapel Hill, NC, United States; Thurston Arthritis Research Center, University of North Carolina at Chapel Hill, Chapel Hill, NC, United States; Department of Cell Biology & Physiology, University of North Carolina at Chapel Hill, Chapel Hill, NC, United States; Lineberger Comprehensive Cancer Center, University of North Carolina at Chapel Hill, Chapel Hill, NC, United States; Curriculum in Genetics & Molecular Biology, University of North Carolina at Chapel Hill, Chapel Hill, NC, United States

## Abstract

**Motivation:**

Deriving biological insights from genomic data commonly requires comparing attributes of selected genomic loci to a null set of loci. The selection of this null set is non-trivial, as it requires careful consideration of potential covariates, a problem that is exacerbated by the non-uniform distribution of genomic features including genes, enhancers, and transcription factor binding sites. Propensity score-based covariate matching methods allow the selection of null sets from a pool of possible items while controlling for multiple covariates; however, existing packages do not operate on genomic data classes and can be slow for large data sets making them difficult to integrate into genomic workflows.

**Results:**

To address this, we developed *matchRanges*, a propensity score-based covariate matching method for the efficient and convenient generation of matched null ranges from a set of background ranges within the Bioconductor framework.

**Availability and implementation:**

Package: https://bioconductor.org/packages/nullranges, Code: https://github.com/nullranges, Documentation: https://nullranges.github.io/nullranges.

## 1 Introduction

Genome-wide analyses can provide valuable insights into biological systems and human disease by revealing patterns of features that may be missed by interrogation of individual loci. Determining if observed trends are statistically significant, however, commonly requires comparing attributes between a focal and a null set of genomic loci. Accurate inference requires that null sets exhibit similar distributions of covariates observed in the focal set, to mitigate interpretability issues due to confounding. This can be challenging since many common covariates (e.g. GC content, gene density, histone acetylation, chromatin accessibility, etc.) are not uniformly distributed throughout the genome and must therefore be explicitly controlled when selecting null sets of loci (Bickel et al. 2010). Propensity score-matching is a computational method that allows for the selection of covariate-matched sets and several packages implement it within the R programming language (Ho et al. 2011; Sekhon 2011). However, these packages can be slow for genome-scale data sets and are not well-integrated into genomic analysis platforms such as Bioconductor making them difficult to incorporate into genomic workflows.

To address this problem, we developed *matchRanges*, an efficient and convenient tool for generating covariate-matched sets of genomic ranges from a pool of background ranges. *matchRanges* computes for each range a propensity score, the probability of assigning a range to focal or background groups, given a chosen set of covariates. It provides three methods including nearest-neighbor matching, rejection sampling, and stratified sampling for null set selection (Ho et al. 2007). Additionally, *matchRanges* provides utilities for accessing matched data, assessing matching quality, and visualizing covariate distributions. The code has been optimized to accommodate genome scale data sets, such that most matchRanges functions can efficiently process sets of millions of loci in seconds on a single core (Supplementary Fig. S1). *matchRanges* accepts and returns common Bioconductor objects, such as *GRanges* and *GInteractions* for seamless integration with existing workflows (Gentleman et al. 2004; Lawrence et al. 2013; Lun et al. 2016) (Supplementary Fig. S2). matchRanges is distributed as part of the *nullranges* package, with multiple software vignettes. *matchRanges* is ideally suited to cases in which feature covariates are known and differ between focal and pool sets. If controlling for local genomic context is of interest, the sister function *bootRanges* may be more appropriate.

## 2 The matchRanges workflow

**Figure 1.**
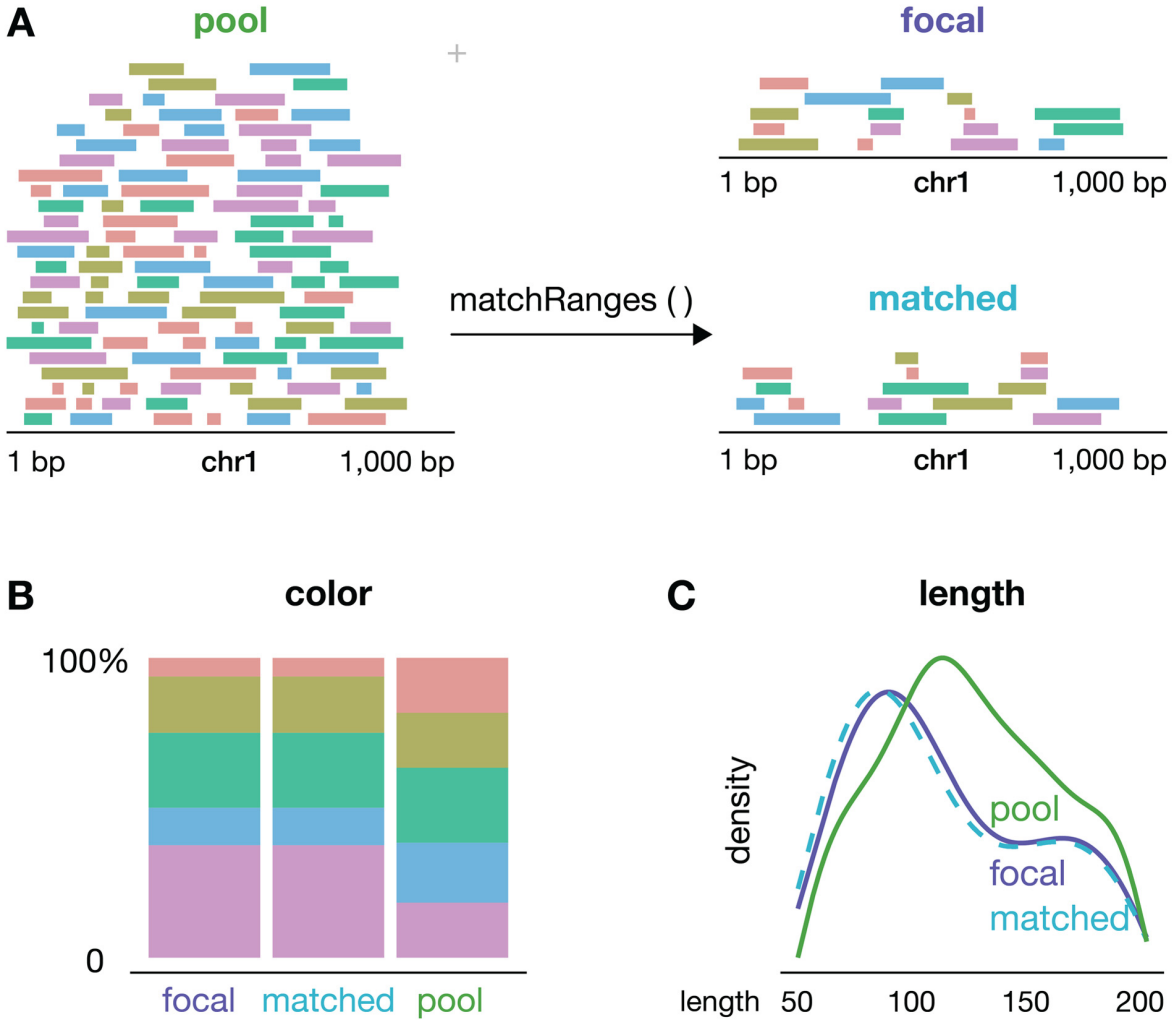
matchRanges workflow. (A) A schematic demonstrating how the *matchRanges* function can be used to select a set of *GRanges* matched for covariate features of color and length. (B and C) Example visualization of covariate distributions for assessing matching quality and covariate balance. Figure generated with the *plotgardener* R/Bioconductor package (Kramer et al. 2022).

To generate a covariate-matched set of ranges, users can provide *data.frame*, *Granges*, or *GInteractions* R objects annotated with columns describing one or more potentially confounding covariates (Lawrence et al. 2013; Lun et al. 2016; Dowle and Srinivasan 2021). The *matchRanges* function takes as input a “focal” set of data to be matched and a “pool” set of background ranges to select from. *matchRanges* performs subset selection based on the provided covariates and returns a null set of ranges with distributions of covariates that approximately match those of the focal set (Fig. 1A). Users should ensure that focal and pool sets share features across all strata being matched to obtain an adequately matched set (Westreich and Cole 2010; Zhu et al. 2021). This allows for an unbiased comparison between features of interest in the focal and matched sets without confounding by matched covariates. As the returned matched sample object is the same class as the inputs, it can be easily incorporated into new or existing Bioconductor workflows (Lee et al. 2019).

A key aspect of inference based on covariate matching is visual inspection of the results. We provide several functions to assess the overall quality of matching, including plots of the distribution of covariates amongst the “focal,” “pool,” and “matched” sets (Fig. 1B and C). Accessor functions allow users to easily extract data for further inspection or integration with covariate balance packages, such as *cobalt* (Greifer 2020) (Supplementary Fig. S3). Since matching is a pre-processing step, multiple matching methods can be tried and assessed before downstream analyses.

Detailed documentation on how to use *matchRanges* and when to use each matching method is available at an accompanying website (https://nullranges.github.io/nullranges), which contains step-by-step tutorials and biological case studies demonstrating the power of *matchRanges*.

## 3 Conclusion

*matchRanges* is a collection of R functions for generating covariate matched ranges to test associations between sets of genomic ranges. Distributed as part of the *nullranges* R package, *matchRanges* uses a propensity score-based method to perform subset selection on genomic ranges, allowing fair comparisons between two sets of interest while avoiding problems with confounding by nuisance covariates. The package provides functions for assessing, visualizing, and extracting matched data that integrates seamlessly into existing Bioconductor workflows. *matchRanges* offers similar matching performance to existing packages but with increased ease of use, scalability, and built-in diagnostic analyses. *matchRanges* will be useful to genomic researchers from all disciplines and will help accelerate scientific progress by improving the accuracy and rigor of genomic analyses.

## Supplementary Material

btad197_Supplementary_Data
