# Supplementary material for: matchRanges: generating null hypothesis genomic ranges via covariate-matched sampling
Source: Bioinformatics. 2023 Apr 21;39(5):btad197. doi: 10.1093/bioinformatics/btad197 (PMC10168584; doi:10.1093/bioinformatics/btad197)
Supplement: btad197_Supplementary_Data [file btad197_supplementary_data.zip › SupplementaryFigureLegends.docx]

**Supplementary Figure 1. matchRanges run time.** Runtime analysis for *matchRanges* and *MatchIt* applied to simulated data. Data were matched for one or two continuous features for each matching method and replacement option. Sample size contains 95% values as pool and 5% as focal. *MatchIt* (v4.5.0) was run with default parameters using method=“nearest”. Benchmarking was performed 10 times using the *microbenchmark* R package on a single core machine (2.50 GHz Intel processor, 16Gb of memory). Median runtime in seconds is shown for evenly spaced datasets spanning 10^3^ to 10^7^ in size for rejection and nearest methods and 10^3^ to 10^6.25^ for matchit and stratified.

**Supplementary Figure 2. Class structure.** Overview of the *matchRanges* class structure and methods. The *Matched* class is combined with either the *DataFrame, data.frame, data.table, GRanges,* or *GInteractions* classes (*left panel*) to create the *MatchedDataFrame*, *MatchedGRanges*, or *MatchedGInteractions* subclasses (*middle panel*). Each subclass behaves as a combination of both its superclasses - with access to both methods of the *Matched* class (*right panel*) and each respective class’ methods.

**Supplementary Figure 3. Assessing covariate balance with *matchRanges* and *cobalt*.** A simulated dataset containing 10^4^ data points (500 focal, 9500 pool) was matched for continuous (feature2) and discrete (feature3) covariates with *MatchIt* and *MatchRanges* using ‘nearest’ matching with replacement. **(A)** Density plots comparing distributions of the continuous “feature2” among the focal, pool, *MatchIt*-matched (n=443), and *matchRanges*-matched (n=500) sets. **(B)** Stacked bar plots comparing proportions of the discrete “feature3” among the focal, pool, *MatchIt*-matched (n=443), and *matchRanges*-matched (n=500) sets. **(C)** Love plot (Love 2004) assessing covariate balance by comparing the mean standardized differences (“ps” and “feature2”) or mean differences (“feature3” strata) when comparing the unadjusted (focal vs. pool) to adjusted (focal vs. *matchRanges* and focal vs. *MatchIt*) sets. “ps” denotes the propensity scores. Values calculated with the “bal.tab()” function from *cobalt*.

**References**

Love, Thomas. 2004. “Graphical Display of Covariate Balance.” *Presentation, See Http://Chrp. Org/Love/JSM2004RoundTableHandout. Pdf* 1364.
